# Effect of a regular consumption of traditional and roasted oat and barley flakes on blood lipids and glucose metabolism–A randomized crossover trial

**DOI:** 10.3389/fnut.2023.1095245

**Published:** 2023-02-02

**Authors:** Sarah Reiners, Sandra Hebestreit, Lisa Wedekind, Michael Kiehntopf, Anne Klink, Silke Rummler, Michael Glei, Stefan Lorkowski, Wiebke Schlörmann, Christine Dawczynski

**Affiliations:** ^1^Junior Research Group Nutritional Concepts, Institute of Nutritional Sciences, Friedrich Schiller University Jena, Jena, Germany; ^2^Competence Cluster for Nutrition and Cardiovascular Health (nutriCARD) Halle-Jena-Leipzig, Jena, Germany; ^3^Institute of Medical Statistics, Computer and Data Sciences, Jena University Hospital, Jena, Germany; ^4^Institute of Clinical Chemistry and Laboratory Diagnostics, Jena University Hospital, Jena, Germany; ^5^Institute of Transfusion Medicine, University Hospital Jena, Jena, Germany; ^6^Department of Nutritional Toxicology, Institute of Nutritional Sciences, Friedrich Schiller University Jena, Jena, Germany; ^7^Department of Nutritional Biochemistry and Physiology, Institute of Nutritional Sciences, Friedrich Schiller University Jena, Jena, Germany

**Keywords:** oat, barley, roasting, β-glucan, blood lipids, cardiovascular risk factors

## Abstract

**Background:**

Regular consumption of the soluble dietary fiber β-glucan is associated with decreased total cholesterol (TC), low-density lipoprotein (LDL) cholesterol and blood glucose. Barley and oat flakes as natural sources of β-glucan were roasted to improve sensory quality. The aim of this study was to investigate whether roasting of barley and oat flakes changes the physiological impact of the β-glucan-rich flakes on glucose and lipid metabolism.

**Method:**

A five-armed randomized crossover trial design was used. The intervention study was conducted from May 2018 to May 2019 and included 32 healthy subjects with moderately increased LDL cholesterol (≥2.5 mmol/L). During the 3-week intervention periods, 80 g of roasted or traditional barley or oat flakes, or four slices of white toast bread per day were consumed for breakfast. At the start and the end of each intervention, fasting and postprandial blood was taken. The intervention periods were separated by 3-week wash-out periods.

**Results:**

During the interventions with the cereal flakes, TC and LDL cholesterol concentrations were significantly reduced compared to baseline values by mean differences of 0.27–0.33 mmol/L and 0.21–0.30 mmol/L, respectively (*p* < 0.05), while high-density lipoprotein (HDL) cholesterol was only reduced after the intervention with barley flakes (*p* < 0.05). After the intervention period with toast, TC and HDL cholesterol increased (*p* < 0.05). The fasting levels of triglycerides, fasting blood glucose and insulin did not change in any group. The effects of traditional and roasted varieties on blood lipids did not differ between the groups.

**Conclusion:**

The regular consumption of traditional or roasted barley and oat flakes contributes to the management of cardiovascular diseases by improving TC and LDL cholesterol.

**Clinical trial registration:**

https://clinicaltrials.gov/ct2/show/NCT03648112, identifier NCT03648112.

## 1. Introduction

Cardiovascular diseases (CVD), cancer, and diabetes mellitus type 2 (DMT2) are three of the four main non-communicable diseases which are responsible for most of premature deaths (1). These chronic diseases can be prevented by improving lifestyle factors including well-balanced, healthy diet rich in dietary fiber (2, 3). The regular consumption of dietary fiber can reduce blood cholesterol as well as postprandial glucose levels and insulin response (4). Numerous studies have shown that especially the consumption of β-glucan from oat and barley may reduce the risk for the development of CVD or DMT2 (5, 6).

Cereal β-glucan is a soluble dietary fiber and high-molecular non-starch polysaccharide comprised of β-(1–4)- and β-(1–3)-linked β-D-glucopyranosyl subunits in varying proportions. Cell walls of the endosperm and aleurone layer of barley and oat grains contain the highest amounts of β-glucan compared to other cereals (7–9). Oat and barley flakes contain an average of 4.6 and 5.2% β-glucan, respectively (10–12). Numerous studies indicated the health-promoting effects of barley and oat and provide the basis for official health claims by the European Food Safety Authority (EFSA) (13–33). Therefore, 4 g β-glucan from oats or barley for each 30 g of available carbohydrates should be consumed per meal to obtain a reduction of the postprandial glucose level (5, 34). For hypercholesterolemic patients it is suggested to consume 3 g of β-glucans from oats and barley per day to achieve a reduction of blood cholesterol and therefore a reduction of risk factors for CVD (34, 35).

These health-promoting effects of β-glucan depends on its physicochemical properties in particularly high molecular weight, viscosity, and solubility (36–42). Some *in vivo* studies showed a lower postprandial glucose (37, 38) and serum cholesterol with increasing viscosity of β-glucan (42).

Despite all these health-promoting effects and the fact that barley belongs to the four most cultivated grains worldwide behind maize, rice, and wheat, only less than one percent of cultivated barley is directly used as food in Europe. The largest amount is used for animal feed and as grain for malting and brewing to produce beer and whiskey. Oats are produced in smaller quantities but 14% of the oats produced in Europe are used as human food (43–46).

Roasting is a process that can increase sensory attractivity and the consumer acceptance of foods by improving texture and flavor of foods like nuts and grains. Schlörmann et al. (10, 11) showed in their studies, that scores for taste, texture, odor and appearance increased along with increasing roasting temperatures obtaining an optimal crispy texture and the typical roasted taste and aroma. However, this heat treatment also involves chemical and structural changes such as the formation of Maillard reaction products which are not only responsible for the typical roasted taste but also for the formation of acrylamide (47, 48). Schlörmann et al. (10, 11) have shown in previous studies that health-related compounds such as protein, dietary fiber and especially β-glucan were not affected by roasting barley at a maximum of 170–180°C and oat at a maximum of 160°C, while the sensory quality was improved. However, the physiological effects of roasted barley and oat products regarding the reduction of glucose and cholesterol still need to be investigated. Therefore, our intervention study was conducted to evaluate the effects of a regular consumption of barley and oat flakes in traditional and roasted form on glucose and lipid metabolism.

## 2. Materials and methods

### 2.1. Subjects

Thirty-two mildly hypercholesterolemic subjects [low-density lipoprotein (LDL) cholesterol: ≥2.5 mmol/L; aged between 25 and 75 years; 22 women, 10 men] from Thuringia (Germany) entered the study after giving their written informed consent. Subjects receiving lipid-lowering medications or glucocorticoids were not included. Those subjects suffering from gastrointestinal or metabolic diseases (e.g., diabetes mellitus), hypercholesteremic patients with previous familial cardio-vascular events, and subjects taking dietary supplements (e.g., fish oil capsules, vitamin E), having been treated in the previous 3 months with fiber-based dietary supplements and/or probiotics and having known food allergies were also excluded. LDL cholesterol was checked during a screening visit by taking a blood sample.

Subjects could be withdrawn from the study at any time after enrollment for the following reasons: at patient’s request, due to serious infections, or if patient compliance with the study protocol was doubtful. The study was conducted in accordance to the Helsinki Declaration of 1975 as revised in 1983. The study protocol was approved by the Ethical Committee of the Friedrich Schiller University Jena (reference number 5503-04/18) and registered by ClinicalTrials.gov(NCT03648112).

### 2.2. Study protocol

**FIGURE 1:**
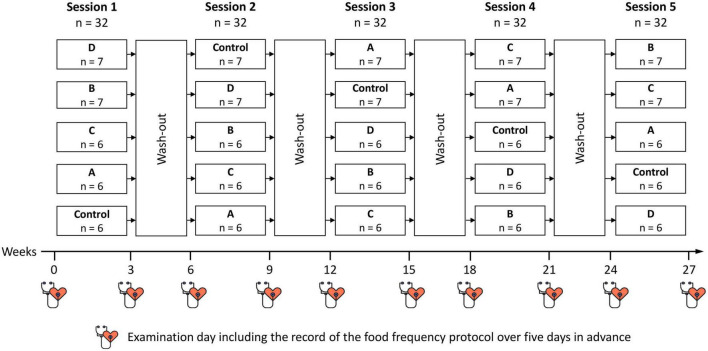
Design of the intervention study. A, roasted oat flakes; B, roasted barley flakes; C, traditional oat flakes; D, traditional barley flakes; Control, white toast bread.

The randomized five-armed crossover study consisted of five 3-week intervention periods with 3-week wash-out periods in between (Figure 1). We used commercially available oat flakes which are also often termed “oatmeal” or “rolled oats” and comparable barley flakes and labeled these flakes as traditional flakes. The traditional flakes were compared with roasted ones and a control intervention. The sequence of testing was counterbalanced over periods using a 5 × 5 Williams-design. The sequence of intervention was always the same. However, there were five groups always beginning with a different intervention. The sequence of the interventions of each group is presented in Figure 1.

**FIGURE 2:**
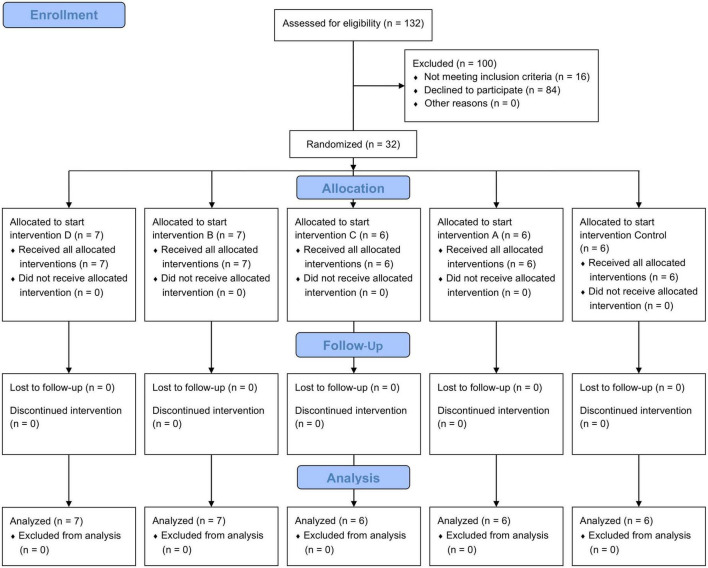
Flowchart diagram of the selection procedure of study participants. 132 subjects were screened for enrollment. Of them, 32 met the inclusion criteria and were randomized into five groups, each of them starting with another intervention. The other 100 subjects were excluded because they did not meet the inclusion criteria or declined to participate. All subjects completed the study and were included in the analysis.

Prior to the study, 132 subjects were screened for enrollment. A total of 32 participants met the inclusion criteria. For assigning the subjects to the first supplement to start with, a randomization list was generated with R studio (Figure 2). The allocation ratio of the study groups was 1:1:1:1:1. By applying the crossover design, all subjects ran through each intervention. The entire net duration of the study was 27 weeks (five 3-weeks interventions plus four 3-weeks wash-out phases in between). The wash out period during Christmas time was extended to 6 weeks to prevent bias by changes in dietary patterns (49).

At the beginning and at the end of each intervention period the subjects visited the study center at the University Hospital of Jena for collection of anthropometric data and taking fasting and postprandial blood samples. The subjects arrived in the morning following a 12-h overnight fasting. Height was only obtained during the first visit of the first period without shoes using a stadiometer (Seca 213, Hamburg, Germany). Body weight was measured using a digital floor scale (BC-313, Tanita Europe B.V., Amsterdam, Netherlands) with all subjects without shoes and waist circumference was determined using a measuring tape for circumference (Seca 201, Hamburg, Germany). Seated, resting blood pressure was obtained after the subject had been seating for at least 10 min and by using an automatic blood pressure device (boso-medicus uno, BOSCH & SOHN, Jungingen, Germany). During the examination, the subjects received a test meal to evaluate postprandial blood glucose and insulin levels. The test meal consisted of the respective intervention supplement and 200 ml whole milk (3.5% total fat). After obtaining the fasting blood samples, the subjects consumed the test meal within 20 min. Next blood samples were taken at 30, 60, 120, and 180 min after the fasting blood sample. During this 3-h examination, subjects were only allowed to drink water. Before the start and at the end of each study period, subjects documented their normal nutritional habits over 5 days in a food frequency protocol (FFP). The FFP originated from the Prodi^®^ version 6.1 software (Nutri-Science, Freiburg, Germany). This protocol includes common foods with usual portion sizes as a check list. Subjects kept this tally sheet over 5 days. The daily energy and nutrient intake was then calculated by the software Prodi^®^.

### 2.3. Study diet

Subjects consumed either 80 g of roasted oat flakes (supplement A), roasted barley flakes (supplement B), traditional oat flakes (supplement C), or traditional barley flakes (supplement D) daily during the intervention periods. The fifth intervention consisted of the control supplement which was four slices of white toast bread (Lieken, Lutherstadt Wittenberg, Germany). Barley flakes were obtained from a special cultivar with low amylose but high β-glucan content (5.0/100 g) (50). Beta-glucan content of oat flakes was 4.0/100 g. Both traditional flakes varieties are commercially available and were provided by Peter Kölln, Elmshorn, Germany (oat flakes) and Dieckmann cereals, Rinteln, Germany, (beta^®^-barley flakes).^1^ These traditional flakes varieties were also used for the roasting process.

Roasting of barley and oat flakes was performed by Probat von Gimborn Maschinenfabrik (Emmerich am Rhein, Germany). Oat flakes were roasted at 150°C and barley flakes 160°C for approx. 20 min. These temperatures were chosen because of previous studies conducted by Schlörmann et al. who investigated the effects of different roasting conditions on the chemical composition and sensory quality of oat and barley products (10, 11). A good sensory quality and acrylamide levels below the benchmark levels of acrylamide in breakfast cereals as defined by the European Commission (150 μg/kg for spelt, maize, oat, barley, and rice-based products) was decisive for the roasting conditions (51).

The four slices of white toast bread provided comparable amounts of energy and total carbohydrates but only 2.8 g fiber per serving (Table 1).

**TABLE 1:** Nutritional values of study products.

	Control: white toast bread (4 slices =̂ 100 g)	Traditional oat flakes (80 g)	Traditional barley flakes (80 g)
Energy [kcal]	265	289	267
Fat [g]	3.8	5.4	1.7
Carbohydrates [g]	48.0	44.8	50.6
Sugars [g]	4.0	1.0	1.4
Dietary fiber [g]	2.8	8.8	10.0
β-glucan [g]	0	3.2	4.1
Protein [g]	8.2	11.2	7.4
Sodium [g]	1.3	<0.01	<0.01

All flakes were offered in neutral packages which were packed by unblinded study staff. The interventions with cereal flakes (traditional vs. roasted) were double-blinded, and the subjects and the blinded researches were not informed about the flakes they received or dispensed. During the intervention periods the subjects received recipes for breakfast for 20 days according to their energy intake (Table 2). Energy intake was determined by using the table for energy intake by the German Nutrition Society (52). The recipes included all 80 g of oat or barley flakes or four slices of white toast bread. They served as suggestions for preparations, but the participants were free to add or change ingredients as long as all flakes or slices of white toast bread were consumed. Further thermic treatment of the flakes and consumption of other β-glucan containing products was prohibited. The FFP and a query how many flakes or toast breads were left over after the intervention period served as intake control of the study products.

**TABLE 2:** Nutritional values of breakfast recipes for three energy levels expressed as mean ± SD.

	Energy level of 1,700 kcal/day		Energy level of 2,200 kcal/day		Energy level of 2,700 kcal/day	
	Flakes recipes	White toast bread recipes	Flakes recipes	White toast bread recipes	Flakes recipes	White toast bread recipes
Energy [kcal]	462 ± 18	458 ± 22	561 ± 15	541 ± 32	660 ± 23	651 ± 42
Fat [g]	9.6 ± 3.2	13.9 ± 3.6	14.3 ± 4.1	18.4 ± 5.0	19.3 ± 4.8	24.4 ± 5.6
Carbohydrates [g]	67.9 ± 7.1	58.1 ± 6.9	76.5 ± 8.9	63.0 ± 10.8	84.6 ± 12.0	68.6 ± 14.5
Sugars [g]	19.2 ± 6.9	13.1 ± 6.6	26.9 ± 8.7	17.6 ± 10.1	34.3 ± 11.4	23.1 ± 13.9
Dietary fiber [g]	11.6 ± 1.6	5.5 ± 1.6	13.1 ± 2.3	6.3 ± 2.2	14.4 ± 2.6	7.2 ± 3.0
Protein [g]	18.2 ± 3.6	21.0 ± 5.8	22.6 ± 4.1	26.3 ± 9.0	26.9 ± 5.7	34.1 ± 12.8

### 2.4. Blood collection and analytical methods

On examination days, subjects arrived at the Institute of Transfusion Medicine, Jena University Hospital at 8:00 h following a 12-h overnight fast. An intravenous catheter (Sarstedt, Nümbrecht, Germany) was fitted into antecubital vein (retrograde) for blood collection. The fasting and postprandial venous blood was collected and centrifuged (10 min, 4°C, 1,300 × *g* and 2,500 × *g*, respectively) for separation of plasma and serum. Study parameters were analyzed immediately after blood sampling at the Institute of Clinical Chemistry and Laboratory Diagnostics, Jena University Hospital. The laboratory analyses were performed by trained personnel to assess total cholesterol (TC), high-density lipoprotein (HDL), LDL cholesterol and triglycerides as primary outcomes as well as biomarkers for diabetes risk (fasting glucose and insulin), high-sensitive c-reactive protein (hs-CRP), and small blood count as secondary outcomes. The analyses of chemical parameters in plasma, serum or whole blood were measured by applying an Abbott Architect CI 16200 analyzer (Abbott, Wiesbaden, Germany), XN 1000 (Sysmex, Norderstedt, Germany), or Tosoh HLC-723G11 (Sysmex) according to the manufacturer’s recommendations at the Institute of Clinical Chemistry and Laboratory Diagnostics, Jena University Hospital (Supplementary Table 1). HOMA-IR was calculated according to the formula: fasting insulin (mU/L) × fasting glucose (mmol/L)/22.5.

### 2.5. Calculations

The postprandial responses over 3 h after a test meal were determined by using the incremental area under the curve (AUC). The AUCs were calculated using the linear trapezoidal rule and the concentration above the fasting values measured at the time points 0, 30, 60, 120, and 180 min.

### 2.6. Statistical analysis

The sample size calculation was based on data previously published by Behall et al. (53, 54) and determined by using a power-analysis (SAS proc power, version 9.4.). In this 5 × 5 Williams-design with four interventions and one control, a mean difference of 0.3 mmol/L was estimated between interventions and control.

For reaching a power of 80% with a significance level of 0.05, six subjects per group and time point were required (*n* total = 30). To consider potential dropouts of 7%, a total of 32 subjects were included.

Outlier detection for each variable was performed by descriptive statistics and histograms. For each variable, the differences between baseline values at the week before starting the intervention (week 0) and values during the last week of the intervention (week 3) were determined by Wilcoxon signed-rank test.

To prevent a possible carry-over effect, the study includes a 3-week wash-out period between the interventions. Nevertheless, for each variable, a pre-test for carry-over was performed prior to assessing treatment efficacy by using the baseline values. There was no statistically significant evidence for carry-over from the comparison of values immediately prior to each intervention period for any parameter.

Analog, the treatment effects were tested using a mixed model with random intercept. For all variables, we calculated the difference between the values from week 3 and the baseline values. Then we tested, if treatment and sequence of interventions have a significant influence. In case of significance, pairwise comparisons of differences (end values minus baseline) were done with Tukey-Kramer correction (55, 56).

Statistical analyses were performed using SAS Enterprise Guide version 9.4 (Cary, NC, USA) and the software R (version 4.1.1; The R Foundation, Indianapolis, IN, USA). All tests were considered significant at *p* < 0.05.

## 3. Results

### 3.1. Subjects and compliance

A total of 32 subjects (men: 10; women: 22; mean age 51.0 years) underwent randomization and started the first of the five 3-weeks interventional periods starting from May 2018, Six or seven participants set up a group and started with the same intervention (Figures 1, 2). No participant dropped out during the whole study period. Until May 2019 all 32 participants had completed all five intervention periods. Table 3 shows the characteristics of the study participants at baseline.

**TABLE 3:** Baseline characteristics of the study subjects expressed as median and interquartile range (IQR).

Study parameters	Baseline *n* = 32
Age [y]	52.0/14.8
Weight [kg]	73.3/16.6
BMI [kg/m^2^]	25.7/4.6
Waist circumference [cm]	91.0/16.4
Systolic blood pressure [mmHg]	125.5/15.0
Diastolic blood pressure [mmHg]	80.5/11.5
TC [mmol/l]	5.52/1.23
Triglycerides [mmol/l]	1.13/0.53
LDL cholesterol [mmol/l]	3.90/1.28
HDL cholesterol [mmol/l]	1.40/0.64
Fasting plasma glucose [mmol/l]	5.60/0.85
HbA_1c_ [%]	5.50/0.40
HOMA-IR	1.49/1.10
hs-CRP [mg/l]	1.20/1.30

BMI, body mass index; hs-CRP, high-sensitive c-reactive protein; HbA_1*c*_, glycated hemoglobin A_1*c*_; HDL, high-density lipoprotein; HOMA-IR, homeostasis model assessment of insulin resistance; LDL, low-density lipoprotein; TC, total cholesterol.

### 3.2. Dietary intake

The study products were completely consumed by all participants. In nine cases, participants reported on flatulence at the begin of the interventions with flakes. The FFPs showed that the participants’ usual diet provided between 1,962 and 2,108 kcal/day, 22–24 g/day dietary fiber, 75–82 g/day proteins, 207–220 g/day carbohydrates, 68–80 g/day fats, with 28–32 g/day saturated fatty acids (SFA), 23–29 g/day monounsaturated fatty acids (MUFA), and 11–14 g/day polyunsaturated fatty acids (PUFA) (Table 4). By consuming the cereal flakes, the intake of dietary fiber increased significantly between 3.63 and 6.47 g/day compared to the baseline diet (A: *p* < 0.001; B: *p* = 0.014; C: *p* = 0.012; D: *p* < 0.001) as well as the intake of carbohydrates (during the intervention with barley flakes; B: *p* = 0.029; D: *p* < 0.001), potassium (during the intervention with oat flakes; A: *p* = 0.009; C: *p* = 0.034), magnesium (A: *p* < 0.001; C: *p* = 0.002; D: *p* = 0.018), and vitamin B12 (during the intervention with traditional oat flakes; C: *p* = 0.034). The energy intake was significantly increased during the intervention with traditional barley flakes (*p* = 0.016) compared to the baseline diet. During the intervention with oat flakes, the intake of sodium (A: *p* = 0.003) and cholesterol (A: *p* = 0.038; C: *p* = 0.036) decreased compared to the baseline diet. In contrast, while consuming the control product, energy intake (*p* < 0.001), the intake of carbohydrates (*p* < 0.001), fat (*p* = 0.006), oleic acid (*p* = 0.028), SFA (*p* < 0.001), MUFA (*p* = 0.014), minerals (*p* = 0.038), and sodium (*p* = 0.032) was increased compared to the baseline diet. These differences resulted in a treatment effect for fat (*p* = 0.016), oleic acid (*p* = 0.042), SFA (*p* = 0.001), MUFA (*p* = 0.029), sodium (*p* < 0.001), and cholesterol (*p* = 0.034) (Table 4).

**TABLE 4:** Consumption of nutrients according to FFP (5 days) in the week before starting the intervention (week 0) and during the last week of the intervention (week 3) expressed as median and IQR (*n* = 32).

Nutrients	Week	Roasted oat flakes (A)			Roasted barley flakes (B)			Traditional oat flakes (C)			Traditional barley flakes (D)			Control			P treatment effect^#^
(*n* = 32)		Median/IQR	*p* *	♢	Median/IQR	*p* *	♢	Median/IQR	*p* *	♢	Median/IQR	*p* *	♢	Median/IQR	*p* *	♢	
Energy [kcal]	0	2,108/824			2,100/573			2,065/856			1,978/778			1,962/801			n.s.
	3	2,135/871	n.s.	a	2,075/713	n.s.	a	2,088/841	n.s.	a	2,101/680	≤0.05	a	2,298/865	≤0.001	a	
Dietary fiber [g]	0	24.3/12.5			22.4/10.9			23.2/11.7			24.5/14.1			23.3/9.3			n.s.
	3	29.0/8.6	≤0.001	a	27.2/12.1	≤0.05	a	29.6/11.5	≤0.05	a	28.1/8.5	≤0.001	a	23.2/11.5	n.s.	a	
Protein [g]	0	80.3/36.8			80.6/37.1			81.2/31.8			75.2/33.5			81.6/34.5			n.s.
	3	82.8/26.2	n.s.	a	76.6/31.3	n.s.	a	92.1/29.4	n.s.	a	79.3/33.3	n.s.	a	87.7/44.7	n.s.	a	
Carbohydrate [g]	0	220.2/100.4			206.7/66.1			211.0/112.8			219.0/82.4			219.9/91.7			n.s.
	3	242.6/83.7	n.s.	a	234.5/73.1	≤0.05	a	237.5/115.9	n.s.	a	259.5/73.1	≤0.001	a	256.7/116.4	≤0.001	a	
Fat [g]	0	79.5/35.8			79.9/29.6			78.4/28.6			68.5/31.7			70.4/35.1			≤0.05
	3	74.3/38.5	n.s.	a, b	67.8/32.7	n.s.	a	77.1/32.7	n.s.	a, b	68.8/38.4	n.s.	a, b	82.5/47.2	≤0.01	b	
Oleic acid [g]	0	23.6/11.8			25.4/10.9			23.2/9.8			21.4/11.4			21.9/13.8			≤0.05
	3	21.9/12.6	n.s.	a, b	21.3/11.1	n.s.	a	23.6/11.2	n.s.	a, b	21.3/12.4	n.s.	a, b	24.9/17.1	≤0.05	b	
Linolenic acid [g]	0	1.3/0.9			1.2/0.6			1.1/0.7			1.2/0.5			1.1/0.7			n.s.
	3	1.1/0.9	n.s.	a	1.0/0.8	n.s.	a	1.0/0.6	n.s.	a	1.0/0.5	n.s.	a	1.2/0.8	n.s.	a	
Linoleic acid [g]	0	11.4/3.7			10.4/6.7			10.7/6.1			9.9/5.4			8.5/6.3			n.s.
	3	9.3/5.4	n.s.	a	8.6/5.4	n.s.	a	10.5/5.3	n.s.	a	7.8/5.1	n.s.	a	10.3/6.1	n.s.	a	
Eicosapentaenoic acid [g]	0	600/90			70/280			40/200			60/260			60/350			n.s.
	3	110/340	n.s.	a	60/140	n.s.	a	120/290	n.s.	a	110/260	n.s.	a	150/250	n.s.	a	
Docosapentaenoic acid [g]	0	30/60			30/90			20/40			40/70			20/50			n.s.
	3	30/60	n.s.	a	30/60	n.s.	a	30/50	n.s.	a	20/40	n.s.	a	30/50	n.s.	a	
Docosahexaenoic acid [g]	0	150/190			180/310			120/170			160/220			130/340			n.s.
	3	170/290	n.s.	a	130/200	n.s.	a	160/210	n.s.	a	130/150	n.s.	a	160/190	n.s.	a	
SFA [g]	0	30.2/20.5			30.5/13.0			32.3/12.4			29.5/11.8			27.8/18.6			≤0.01
	3	31.1/14.3	n.s.	a	30.1/15.0	n.s.	a	29.4/13.8	n.s.	a	27.9/17.2	n.s.	a, b	33.7/16.7	≤0.001	b	
MUFA [g]	0	26.0/13.2			28.7/11.1			25.9/14.5			23.4/11.6			24.1/16.1			≤0.05
	3	24.6/14.3	n.s.	a, b	24.8/12.2	n.s.	a	27.2/10.9	n.s.	a, b	24.5/12.8	n.s.	a, b	28.6/18.4	≤0.05	b	
PUFA [g]	0	13.8/5.1			12.2/6.6			12.7/7.5			12.3/7.2			10.5/6.7			n.s.
	3	11.6/5.5	n.s.	a	10.9/6.0	n.s.	a	13.2/5.9	n.s.	a	9.7/8.4	n.s.	a	13.2/7.2	n.s.	a	
Minerals [g]	0	19.1/5.8			18.2/5.1			17.6/6.0			16.9/6.2			17.3/6.0			n.s.
	3	17.2/5.1	n.s.	a	17.5/7.4	n.s.	a	17.6/6.4	n.s.	a	16.2/7.2	n.s.	a	19.5/6.0	≤0.05	a	
Potassium [mg]	0	3,021/959			3,422/1,279			3,170/1,356			3,231/1,363			3,297/1,229			n.s.
	3	3,743/1,400	≤0.01	a	3,025/993	n.s.	a	3,557/1,509	≤0.05	a	3,178/1,113	n.s.	a	3,397/2,174	n.s.	a	
Magnesium [mg]	0	344.2/134.6			343.8/102.8			350.4/166.1			352.3/143.7			329.8/123.5			n.s.
	3	437.1/97.6	≤0.001	a	370.7/118.3	n.s.	a	429.5/127.2	≤0.01	a	376.8/120.3	≤0.05	a	336.7/189.2	n.s.	a	
Sodium [mg]	0	2,365/978			2,032/820			1,981/934			1,932/1,061			2,252/1,415			≤0.01
	3	1,983/1,232	≤0.01	a	1,782/1,040	n.s.	a, b	1,786/955	n.s.	a, b	1,844/1,014	n.s.	a, b	2,376/942	≤0.05	b	
Cholesterol [mg]	0	355.7/257.4			315.8/155.9			309.1/224.9			300.5/162.7			277.4/173.3			≤0.05
	3	279.8/180.2	≤0.05	a	291.6/216.7	n.s.	a	236.0/150.8	≤0.05	a	281.8/247.7	n.s.	a	316.9/228.5	n.s.	a	
Vitamin B_12_ [μg]	0	4.5/2.8			4.5/3.2			3.6/2.7			4.8/3.3			4.8/2.9			n.s.
	3	4.9/3.1	n.s.	a	4.5/3.6	n.s.	a	5.1/3.5	≤0.05	a	5.0/3.5	n.s.	a	5.1/3.8	n.s.	a	

*Significance of comparing week 0 and week 3 within the same group as determined by Wilcoxon signed-rank test.

^♢^Interventions without a common letter are significantly different by Tukey’s test, *p* ≤ 0.05.

^#^Significance of main effect of treatment by mixed model with random intercept. MUFA, monounsaturated fatty acids; n.s., not significant (*p* > 0.05); PUFA, polyunsaturated fatty acids; SFA, saturated fatty acids.

### 3.3. Anthropometric parameters

After the intervention with roasted barley flakes (B), body weight (*p* = 0.028) and body mass index (BMI) were reduced compared to baseline (*p* = 0.035; Table 5). Moreover, the systolic and diastolic blood pressure decreased also by 4.6% (*p* = 0.041) and 6.7% (*p* = 0.035), respectively. The further treatments did not have any effects on the anthropometric parameters, and there was no significant effect between the periods (Table 5).

**TABLE 5:** Anthropometric parameters at baseline (week 0) and at the end of the intervention periods (week 3) expressed as median and IQR (*n* = 32).

Anthropometric parameters		Week	Roasted oat flakes (A)			Roasted barley flakes (B)			Traditional oat flakes (C)			Traditional barley flakes (D)			Control			P treatment effect^#^
(*n* = 32)			Median/IQR	*p* *	♢	Median/IQR	*p* *	♢	Median/IQR	*p* *	♢	Median/IQR	*p* *	♢	Median/IQR	*p* *	♢	
BMI [kg/m^2^]		0	26.0/5.2			25.8/5.3			25.5/4.6			25.8/4.4			25.7/4.3			n.s.
		3	25.8/5.0	n.s.		25.6/4.8	≤0.05		25.7/5.3	n.s.		25.6/5.0	n.s.		25.9/4.2	n.s.		
	Change from baseline [%]		0.1/1.7		a	−0.5/1.3		a	0.3/1.0		a	−0.3/1.5		a	0.2/1.3		a	
Weight [kg]		0	74.4/15.9			75.1/15.1			74.6/16.2			75.2/14.3			74.2/15.6			n.s.
		3	75.1/16.3	n.s.		74.8/14.1	≤0.05		75.1/15.8	n.s.		75.4/15.9	n.s.		73.8/15.8	n.s.		
	Change from baseline [%]		0.1/1.7		a	−0.5/1.4		a	0.3/1.0		a	−0.3/1.5		a	0.2/1.2		a	
Waist circumference [cm]		0	90.3/15.4			91.0/14.0			91.3/15.5			90.0/13.9			89.0/15.0			n.s.
		3	89.8/17.9	n.s.		89.5/13.6	n.s.		89.0/13.5	n.s.		90.0/14.4	n.s.		88.3/11.4	n.s.		
	Change from baseline [%]		−0.7/3.6		a	−0.8/4.4		a	−0.5/4.6		a	0.0/3.5		a	−0.6/3.4		a	
Systolic blood pressure [mmHg]		0	121.0/14.3			130.0/14.8			122.5/16.8			123.5/14.3			122.0/17.5			n.s.
		3	120.5/15.3	n.s.		124.0/12.3	≤0.05		124.0/12.3	n.s.		123.5/11.3	n.s.		125.0/14.0	n.s.		
	Change from baseline [%]		−0.8/9.5		a	−2.2/8.1		a	−0.4/14.3		a	−1.2/8.2		a	1.2/14.5		a	
Diastolic blood pressure [mmHg]		0	74.0/12.0			82.5/14.0			77.0/10.8			78.0/12.8			80.5/13.0			n.s.
		3	78.5/10.8	n.s.		77.0/16.8	≤0.05		79.5/10.5	n.s.		79.5/16.0	n.s.		78.0/12.0	n.s.		
	Change from baseline [%]		2.1/11.3		a	−4.9/12.9		a	0.6/10.2		a	0.6/10.0		a	−0.6/13.8		a	

*Significance of comparing week 0 and week 3 within the same group as determined by Wilcoxon signed-rank test. ^♢^Interventions without a common letter are significantly different by Tukey’s test, *p* ≤ 0.05. ^#^Significance of main effect of treatment by mixed model with random intercept. BMI, body mass index; n.s., not significant (*p* > 0.05).

### 3.4. Fasting biochemical parameters

During all interventions with oat and barley flakes, TC and LDL cholesterol were reduced by in average 4.8% for TC (A: *p* = 0.004; B: *p* = 0.015; C: *p* = 0.006; D: *p* = 0.006) and 6.5% for LDL cholesterol (A: *p* = 0.002; B: *p* = 0.009; C: *p* = 0.012; D: *p* = 0.004), respectively, compared to baseline (Table 6). In contrast, TC increased by 3.9% (*p* = 0.012) in the control period. The regular consumption of traditional and roasted barley flakes was associated with a reduction of HDL cholesterol by 4.8% (*p* = 0.049) and 5.4% (*p* = 0.001), respectively (Table 6). After the control intervention, HDL cholesterol increased by 3.7% (*p* = 0.012). This resulted in a treatment effect for HDL cholesterol (*p* < 0.001). Moreover, there was a treatment effect for TC (*p* < 0.001) and LDL cholesterol (*p* = 0.004) as these parameters decreased significantly due to consumption of oat and barley flakes (Table 6). Triglycerides were not affected by any intervention.

**TABLE 6:** Fasting biochemical parameters at baseline (week 0) and at the end of the intervention periods (week 3) expressed as median and IQR (*n* = 32).

Biochemical parameters		Week	Roasted oat flakes (A)			Roasted barley flakes (B)			Traditional oat flakes (C)			Traditional barley flakes (D)			Control (K)			P treatment effect^#^
(*n* = 32)			Median/IQR	*p* *	♢	Median/IQR	*p* *	♢	Median/IQR	*p* *	♢	Median/IQR	*p* *	♢	Median/IQR	*p* *	♢	
**Blood lipids**																		
TC [mmol/l]		0	5.78/1.29			6.03/1.17			5.77/0.79			5.70/1.11			5.82/1.41			≤0.001
		3	5.67/1.22	≤0.01		5.72/1.05	≤0.05		5.74/0.79	≤0.01		5.65/1.39	≤0.01		5.96/0.99	≤0.05		
	Change from baseline [%]		−6.6/11.8		a	−3.6/8.5		a	−4.0/11.5		a	−4.90/10.8		a	3.90/9.6		b	
HDL cholesterol [mmol/l]		0	1.48/0.57			1.42/0.49			1.51/0.65			1.40/0.56			1.44/0.62			≤0.001
		3	1.38/0.69	n.s.		1.41/0.49	≤0.001		1.50/0.62	n.s.		1.42/0.59	≤0.05		1.47/0.60	≤0.05		
	Change from baseline [%]		−3.0/10.8		a	−5.3/9.5		a	−1.8/10.4		a, b	−4.8/14.9		a	3.7/11.5		b	
LDL cholesterol [mmol/l]		0	3.74/1.06			4.00/1.39			3.77/1.05			3.65/1.15			3.84/1.10			≤0.01
		3	3.51/0.93	≤0.01		3.59/1.05	≤0.01		3.59/1.05	≤0.05		3.52/1.12	≤0.01		3.77/0.95	n.s.		
	Change from baseline [%]		−9.10/11.6		a	−4.40/12.2		a	−5.60/12.1		a	−6.90/10.4		a	2.60/14.1		b	
Triglycerides [mmol/l]		0	1.10/0.48			1.07/0.60			1.16/0.58			1.12/0.65			1.17/0.55			n.s.
		3	1.08/0.66	n.s.		1.21/0.53	n.s.		1.19/0.64	n.s.		1.13/0.68	n.s.		1.25/0.72	n.s.		
	Change from baseline [%]		0.0/32.8		a	9.1/45.3		a	0.1/44.9		a	3.8/23.9		a	11.3/32.8		a	
**Diabetes risk**																		
Glucose [mmol/l]		0	5.60/0.67			5.50/0.73			5.60/0.70			5.55/0.55			5.50/0.60			n.s.
		3	5.50/0.70	n.s.		5.54/0.62	n.s.		5.40/0.32	≤0.1		5.50/0.62	n.s.		5.40/1.00	n.s.		
	Change from baseline [%]		0.0/7.1		a	0.0/6.8		a	−2.5/9.3		a	−1.8/9.5		a	−1.8/7.5		a	
Insulin [mU/l]		0	5.95/4.02			6.20/4.93			6.35/3.50			5.70/4.25			6.70/4.23			n.s.
		3	7.20/4.18	n.s.		6.15/3.68	n.s.		6.60/3.62	n.s.		6.15/4.93	n.s.		6.65/4.12	n.s.		
	Change from baseline [%]		2.9/38.7		a	−4.1/78.5		a	4.1/44.2		a	1.9/57.4		a	−7.5/56.5		a	
HbA_1c_ [%]		0	5.40/0.40			5.40/0.42			5.50/0.42			5.45/0.40			5.35/0.30			n.s.
		3	5.50/0.40	n.s.		5.50/0.42	n.s.		5.40/0.42	n.s.		5.40/0.33	n.s.		5.30/0.40	n.s.		
	Change from baseline [%]		0.0/3.7		a	0.0/1.9		a	0.0/1.8		a	0.0/1.8		a	0.0/1.9		a	
HOMA-IR		0	1.58/1.17			1.44/1.22			1.60/1.01			1.42/1.22			1.67/1.18			n.s.
		3	1.77/1.38	n.s.		1.54/0.94	n.s.		1.64/0.88	n.s.		1.48/1.25	n.s.		1.66/1.30	n.s.		
	Change from baseline [%]		−0.5/40.4		a	−3.4/92.4		a	1.5/43.2		a	0.9/65.8		a	−8.8/59.5		a	
**Inflammation**																		
High sensitive C-reactive protein [mg/l]		0	0.85/1.07			0.75/0.92			1.00/1.03			0.80/1.45			0.80/1.05			n.s.
		3	0.80/1.00	n.s.		1.00/1.15	n.s.		0.75/1.25	n.s.		0.80/1.02	n.s.		1.10/0.90	n.s.		
	Change from baseline [%]		0.0/35.8		a	7.0/67.5		a	0.0/54.4		a	0.0/42.2		a	0.0/23.6		a	
**Immune and clotting system**																		
Leucocytes [Gpt/l]		0	5.10/1.75			4.95/1.50			4.95/1.90			5.40/1.30			5.10/1.62			≤0.05
		3	5.20/1.88	n.s.		5.00/2.00	n.s.		5.05/1.58	n.s.		4.85/1.88	≤0.05		4.95/2.02	n.s.		
	Change from baseline [%]		1.7/23.2		a, b	0.0/20.5		a	1.8/22.1		a, b	−7.2/19.0		b	2.1/15.7		a, b	
Thrombocytes [Gpt/l]		0	245.0/63.5			245.0/63.3			248.5/51.8			245.5/61.8			242.0/71.3			n.s.
		3	245.5/75.0	n.s.		241.0/53.0	n.s.		239.0/82.3	n.s.		243.0/71.0	≤0.01		236.5/49.5	n.s.		
	Change from baseline [%]		1.2/15.5		a	−1.0/13.8		a	1.0/11.6		a	−4.5/9.1		a	−1.8/10.7		a	
Erythrocytes [Tpt/l]		0	4.60/0.60			4.70/0.50			4.70/0.50			4.70/0.50			4.60/0.60			n.s.
		3	4.60/0.60	n.s.		4.65/0.50	n.s.		4.65/0.53	n.s.		4.70/0.52	n.s.		4.70/0.50	≤0.05		
	Change from baseline [%]		−1.9/7.7		a	0.0/6.3		a	0.0/6.2		a	−2.1/7.7		a	2.2/5.8		a	
Hematocrit [%]		0	0.40/0.03			0.40/0.05			0.40/0.05			0.40/0.04			0.40/0.05			n.s.
		3	0.39/0.03	n.s.		0.41/0.03	n.s.		0.40/0.04	n.s.		0.40/0.04	≤0.05		0.39/0.03	≤0.05		
	Change from baseline [%]		0.0/7.1		a, b	0.0/5.2		a, b	0.0/6.1		a, b	−2.4/7.5		a	1.1/4.8		b	
MCH [fmol]		0	1.79/0.11			1.79/0.11			1.80/0.10			1.79/0.12			1.81/0.11			n.s.
		3	1.81/0.11	n.s.		1.79/0.12	n.s.		1.80/0.11	≤0.1		1.81/0.10	n.s.		1.79/0.12	n.s.		
	Change from baseline [%]		0.6/1.9		a	0.0/2.9		a	−0.3/1.8		a	0.5/1.8		a	−0.5/2.0		a	
MCHC [mmol/l]		0	20.80/0.95			21.00/0.92			21.00/0.60			20.90/0.83			20.90/0.73			n.s.
		3	21.10/0.97	n.s.		20.90/0.70	n.s.		20.80/0.82	n.s.		21.10/0.70	n.s.		20.95/0.72	n.s.		
	Change from baseline [%]		0.5/1.9		a	−0.2/3.1		a	0.0/2.2		a	0.0/2.5		a	0.0/2.5		a	
MCV [fl]		0	86.00/5.50			85.00/5.25			86.00/6.00			86.50/6.25			86.00/6.00			n.s.
		3	85.50/5.25	n.s.		85.50/6.25	n.s.		85.00/7.00	n.s.		86.00/6.00	n.s.		85.50/6.00	n.s.		
	Change from baseline [%]		0.0/2.3		a	0.0/1.4		a	0.0/1.2		a	0.0/2.4		a	0.0/2.7		a	
RDW [%]		0	12.90/0.95			12.90/0.65			13.00/0.80			12.75/0.72			13.10/0.65			≤0.05
		3	12.85/0.85	n.s.		12.85/0.80	≤0.05		12.80/0.90	≤0.1		12.95/0.83	≤0.05		13.10/0.67	n.s.		
	Change from baseline [%]		0.0/3.1		a, b	−0.8/3.1		a	−0.8/1.8		a, b	0.8/3.4		b	0.0/2.0		a, b	
Hemoglobin [mmol/l]		0	8.30/0.95			8.45/ 0.78			8.35/1.03			8.45/0.83			8.25/0.98			n.s.
		3	8.20/0.80	n.s.		8.40/0.85	n.s.		8.40/1.08	n.s.		8.20/1.00	≤0.05		8.30/0.92	≤0.1		
	Change from baseline [%]		0.0/6.2		a	−1.1/6.9		a	−1.1/5.9		a	−2.4/7.5		a	1.2/4.5		a	

*Significance of comparing week 0 and week 3 within the same group as determined by Wilcoxon signed-rank test.

^♢^Interventions without a common letter are significantly different by Tukey’s test, *p* ≤ 0.05.

^#^Significance of main effect of treatment by mixed model with random intercept.

HbA_1*c*_, glycated hemoglobin A_1*c*_; HDL, high-density lipoprotein; HOMA-IR, homeostasis model assessment of insulin resistance; LDL, low-density lipoprotein; MCH, mean corpuscular hemoglobin; MCHC, mean corpuscular hemoglobin concentration; MCV, mean cell volume; n.s., not significant (*p* > 0.05); RDW, red blood cell distribution width; TC, total cholesterol.

Except a trend for a reduction of fasting glucose after the intervention with traditional oat flakes (*p* = 0.083), the analyzed diabetes risk markers and hs-CRP were not influenced by any intervention (Table 6).

For blood count, there were differences between the interventions with roasted and traditional barley flakes observed for leukocytes and red blood cell distribution width (RDW) (*p* = 0.028 and *p* = 0.019, respectively). Other parameters [leukocytes (*p* = 0.046), thrombocytes (*p* = 0.008), hematocrit (*p* = 0.022), and hemoglobin (*p* = 0.025)] decreased in response to the regular consumption of traditional barley flakes whereas the concentration of erythrocytes (*p* = 0.041) and hematocrit (*p* = 0.026) increased in the control period (Table 6).

### 3.5. Postprandial kinetics

Insufficient volume of blood was collected for the determination of glucose in two cases and for the determination of other parameters (TC, triglycerides, LDL and HDL cholesterol) in one case, respectively. This resulted in different group sizes. During the intervention with traditional oat flakes, the AUC of glucose was significantly reduced [median/IQR: 1,045.5/205.9 to 1,005.0/156.4 (mmol/L) × min; *p* = 0.018, Table 7]. The AUC of insulin and triglycerides was not affected by any intervention (Table 7). The AUC of TC and LDL cholesterol was significantly reduced during all interventions with cereal flakes between 3.4–6.7% (TC; A: *p* = 0.006; B: *p* < 0.001; C: *p* = 0.005; D: *p* = 0.007) and 3.4–9.6% (LDL; A: *p* = 0.001; B: *p* = 0.021; C: *p* = 0.006; D: *p* = 0.005) (Table 7), resulting in a significant treatment effect (*p* < 0.001 and *p* = 0.019, Table 7). The AUC of HDL cholesterol significantly decreased during the intervention with roasted barley flakes by 12.75/35.85 (mmol/L) × min (*p* < 0.001). In contrast, the AUC of HDL cholesterol significantly increased during the intervention with toast bread by 10.50/33.04 (mmol/L) × min (*p* = 0.032). For both interventions the intervention effect was significant (*p* < 0.001, Table 7).

**TABLE 7:** Area under the curve (AUCs) at baseline (week 0) and at the end of the intervention periods (week 3) expressed as median and IQR (*n* = 31).

Parameter	Week	Roasted oat flakes (A)			Roasted barley flakes (B)			Traditional oat flakes (C)			Traditional barley flakes (D)			Control			P treatment effect^#^
		Median/IQR	*p* *	♢	Median/IQR	*p* *	♢	Median/IQR	*p* *	♢	Median/IQR	*p* *	♢	Median/IQR	*p* *	♢	
AUC glucose [(mmol/l) × min]	0	1,024.5/184.5			1,035.0/182.3			1,045.5/205.9			1,019.3/233.3			1,005.0/191.3			n.s.
	3	1,020.0/178.5	n.s.		1,004.3/116.6	n.s.		1,005.0/156.4	≤0.05		1,044.3/207.4	n.s.		1,021.5/226.5	n.s.		
Change from baseline [%]		−1.3/8.9		a	−0.3/17.0		a	−4.9/13.3		a	1.6/11.2		a	−1.6/11.4		a	
AUC insulin [(mmol/l) × min]	0	4,746.8/4,257.8			3,647.3/3,013.5			4,470.0/4,161.4			3,273.8/3,056.6			4,746.8/3,624.0			n.s.
	3	4,352.3/3,865.5	n.s.		3,916.5/3,157.1	n.s.		4,068.0/3,190.9	n.s.		3,478.5/3,202.5	n.s.		4,011.0/3,838.1	n.s.		
Change from baseline [%]		0.2/38.7		a	2.7/26.4		a	−9.4/23.4		a	1.9/24.7		a	−1.6/40.0		a	
AUC TC [(mmol/l) × min]	0	1,029.7/193.2			1,070.5/191.3			1,004.9/129.8			1,011.3/197.6			1,046.9/208.2			≤0.001
	3	1,005.9/175.7	≤0.01		1,015.8/188.1	≤0.001		1,010.8/143.3	≤0.01		988.8/201.2	≤0.01		1,026.8/162.7	≤0.1		
Change from baseline [%]		−6.7/8.7		a	−3.6/6.7		a	−3.4/11.5		a	−5.5/9.4		a	3.6/10.4		b	
AUC triglycerides [(mmol/l) × min]	0	198.3/108.5			195.1/134.6			235.3/110.2			214.5/97.8			218.9/119.7			n.s.
	3	213.8/124.7	n.s.		216.0/98.1	n.s.		230.0/125.0	n.s.		222.2/115.1	n.s.		245.6/124.9	n.s.		
Change from baseline [%]		2.3/31.8		a	7.8/45.1		a	2.8/45.4		a	3.4/19.3		a	6.3/34.7		a	
AUC LDL cholesterol [(mmol/l) × min]	0	665.4/191.0			698.0/234.7			652.7/176.9			656.8/196.3			680.8/185.1			≤0.05
	3	628.1/154.7	≤0.001		623.1/197.2	≤0.05		644.5/168.3	≤0.01		647.0/187.1	≤0.01		652.3/201.2	n.s.		
Change from baseline [%]		−9.6/13.1		a	−3.4/15.0		a, b	−4.7/15.0		a	−7.0/9.6		a, b	2.5/14.5		b	
AUC HDL cholesterol [(mmol/l) × min]	0	266.0/102.5			254.3/104.4			264.0/110.1			256.6/112.8			247.8/99.4			≤0.001
	3	249.3/113.7	≤0.1		248.6/90.6	≤0.001		273.1/116.6	≤0.1		245.1/89.7	≤0.1		252.5/111.6	≤0.05		
Change from baseline [%]		−2.5/10.6		a, b	−5.9/11.8		a	−3.2/8.2		a, b	−2.8/10.0		a, b	4.0/12.6		b	

*Significance of comparing week 0 and week 3 within the same group as determined by Wilcoxon signed-rank test.

^♢^Interventions without a common letter are significantly different by Tukey’s test, *p* ≤ 0.05.

^#^Significance of main effect of treatment by mixed model with random intercept. AUC, area under the curve; HDL, high-density lipoprotein; LDL, low-density lipoprotein; n.s., not significant (*p* > 0.05); TC, total cholesterol.

## 4. Discussion

In a sample of mildly hypercholesterolemic subjects, the regular consumption of 80 g traditional or roasted oat or barley flakes for breakfast per day resulted in a significant reduction of TC and LDL cholesterol. The interventions mostly failed to detect any significant changes in the analyzed biomarkers for diabetes and hs-CRP. The data from the postprandial kinetics reflect the results after a regular consumption of the investigated cereal flakes over 3 weeks (Tables 6, 7). In all interventions with cereal flakes, fasting glucose showed a decreasing trend, whereby the regular consumption of traditional oat flakes seemed to induce a slightly stronger effect than the other cereal flakes.

Similar results on TC, LDL cholesterol and on glucose metabolism were also obtained during the BELT study which investigated the effect of 3 g/day oat β-glucan on lipid profile, glycemia and intestinal health in 83 Italian with moderate hypercholesterolemia (LDL cholesterol: ≥3.36 mmol/L) (57). In the 8-week study period the participants received a proprietary formulation of β-glucans. LDL cholesterol concentrations were reduced by 12.2% from baseline after 4 weeks of supplementation and by 15.1% after 8 weeks of supplementation. TC and non-HDL cholesterol were also significantly reduced during both follow-up visits. With 4–9%, the relative LDL cholesterol reduction was smaller in the present study. This fact might be attributed by the shorter intervention period and the higher variability of LDL cholesterol concentrations at baseline. In addition, participants with higher LDL cholesterol concentrations at baseline were enrolled in the BELT study (≥3.36 and ≤4.91 mmol/L). However, the mean reduction of TC and LDL cholesterol by 0.29 and 0.24 mmol/L respectively, is exact in the range of further studies which investigated the cholesterol-lowering effects of ≥3 g oat β-glucan (42, 58, 59).

The effect of oat and barley β-glucan on HDL cholesterol is not conclusive as the results show trends for a reduction that are only significant for the intervention with barley flakes (Tables 6, 7). This is also in line with the meta-analysis of Whitehead et al. (57) and study of Xu (42, 60) who showed no effect on HDL cholesterol or a significant heterogeneity among studies (57).

Our findings on fasting plasma glucose are also in accordance with the results from the BELT study. Like in our study, euglycemic participants were enrolled in the BELT study (57). Meta-analyses already showed that the intake of oat β-glucan is more effective in subjects with DMT2 (61, 62). Nevertheless, we observed a slight reduction for the AUC of glucose after the regular consumption of traditional oat flakes (*p* = 0.018; Table 7). Stronger effects were presented by Wolever et al. (63), as a significant dose-response effect of low amounts of oat β-glucan on glycemic response was also shown. Thereby, each gram of oat β-glucan reduced AUC by 7%. Nevertheless, for a significant reduction in the AUC of glucose, 2.77 g β-glucan per 16.2 g available carbohydrates were necessary which is in accordance with the EFSA health claim (34).

The study interventions with oat and barley flakes had no effects on triglycerides. This is in line with findings of several other studies, as was shown in a meta-analysis of randomized controlled trials by Whitehead et al. (58). The authors reported that adding ≥3 g/day oat β-glucan to the diet reduces LDL cholesterol and TC without changing triglycerides. However, there are hints that β-glucan might reduce triglycerides in healthy people with a normal lipid profile, but also in overweight people or those with diabetes or metabolic syndrome. In a recent systematic review six out of 17 included studies reported significant improvements in triglyceride concentrations (64). However, often higher doses, longer study duration, and the combination with long-chain omega-3 fatty acids or calorie reduction resulted in these stronger effects (64).

Different physicochemical properties of β-glucan can also result in varying study results in human trails (16, 65). Water-solubility of dietary fiber is already known to positively affect serum cholesterol as highly water-soluble β-glucan is more effective in reducing serum cholesterol than poorly water-soluble β-glucan (16, 65). Moreover, especially soluble fiber have a functional relationship with the gut microbiota in the large intestine (66) by exerting a prebiotic effect on the gut microbiota which in turn can regulate cholesterol homeostasis in the host (67, 68). β-Glucan solubility depends on its molecular fine structure, which is different in oat and barley because of different ratios of cellotriosyl to cellotetraosyl units (69). Several studies already demonstrated that barley β-glucans show lower solubility than oat β-glucans (69–71). In the present study, these effects can be evidenced by the mean differences before and after the interventions in the LDL cholesterol AUCs. These were slightly greater (but not significantly) in oat flakes [roasted: −55 (mmol/L) × min; traditional: −61 (mmol/L) × min] than in barley flakes [roasted: −38 (mmol/L) × min, traditional: −35 (mmol/L) × min]. The fact that these differences were not reflected in the fasting concentrations is indicative for a faster postprandial LDL cholesterol clearance after a time of adaptation to an oat-rich diet which is necessary when microbiota need to adapt (72). However, these mechanisms need to be more evaluated and further studies with especially barley β-glucan are warranted.

A further important determinant of the physicochemical characteristics of β-glucan is its molecular weight. The molecular weight of β-glucan affects its water-solubility and viscosity which are responsible for its hypocholesterolemic and hypoglycemic properties (73–75). The molecular weight of barley β-glucan is around 0.15–2.5 × 106 g/mol and of oat β-glucan around 0.065–3.0 × 106 g/mol (76), and can vary due to the diversity in its source, extraction protocol, and methodology employed for its determination (75). In the present study, roasted oat flakes seemed to show a higher AUC of insulin compared to traditional and roasted barley flakes (Table 7). A significant reduction of insulin AUCs only by barley (*p* < 0.005), and not by oat, was also found in a study by Behall et al. (24). They used barley of a special cultivar (Prowashonupana cultivar) with a very high β-glucan content (15 g/100 g) so that the β-glucan content of the barley products was no longer comparable to the oat products (3.23 g β-glucan for oat test meal vs. 12.1 g β-glucan for barley test meal). The higher concentration and a different molecular weight of β-glucan could be responsible for the greater reduction in insulin AUC (61, 70). In contrast, Hallfrisch et al. (17) found no differences between barley and oat on glucose and insulin responses on 22 non-diabetic men and women who consumed 1 g/kg body weight of carbohydrate as glucose or 0.33 g/kg of body weight as oat bran, barley flour, oat or barley extract in a Latin square design after consuming controlled diets for 3 days. AUCs of glucose and insulin responses to oats, barley, and both extracts, were significantly lower than responses to the glucose solution (17). However, the sources of β-glucan were no flakes, what can be again indicative for different physicochemical properties.

Ultimately, the already discussed characteristics, water-solubility, and molecular weight, affect the viscosity. There is evidence, that an increasing solubility and molecular weight of β-glucan is accompanied with higher viscosity. An increased viscosity in the gastrointestinal tract delays the gastric emptying as well as the starch digestion, that in turn, led to reduced postprandial hyperglycemia. The particular microenvironment with high viscosity in the small intestine acts as a physical barrier and prevents the absorption and reabsorption of cholesterol and bile acids, so that more bile acids are excreted which promotes the *de novo* synthesis of bile acids from cholesterol leading to reduced serum cholesterol concentrations (77, 78). In a study, which measured bile acid excretion within 24 h of β-glucan consumption, it revealed that this is increased. Entrapment of whole micelles in the gut owing to higher viscosity is held to be responsible for this effect (79). Therefore, the viscosity is an important determinant of the postprandial glucose regulation and cholesterol lowering effect of β-glucan (37, 38, 42, 65, 67). However, viscosity is further influenced by the concentration of β-glucan and the surrounding food matrix, and these factors can be altered again by food processing and storage (39, 42, 80, 81). The flakes used in this study were already examined on their viscosity by Schlörmann et al. (10, 11). For both flakes the viscosity significantly decreased with roasting. However, regarding postprandial glucose regulation or cholesterol levels, we observed no differences between the roasted and the traditional flakes. As discussed by Schlörmann et al. (11) the reduced viscosities from 255 to 98 mPas may not interfere with these physiological effects. This fact agrees with the studies by Wood et al. (32, 82) and a review by Lazaridou and Biliaderis (74), whereby a huge range of viscosities from 20 to 5,000 mPas is able to improve postprandial glucose and insulin responses. Thus, the gentle treatment of roasting used for our flakes did not have an impact on the physiological effects but improved the sensory quality (10, 11).

Next to the different properties of β-glucans the underlying mechanisms reflected on serum metabolomics linking β-glucan intake and the reduction of TC and LDL cholesterol are under investigation. Therefore, Xu et a. also conducted a study involving healthy adults with mild hypercholesterolemia consuming 80 g oats daily and implemented an untargeted serum metabolomics. They could reveal underlying metabolic pathways such as glycerophospholipids, alanine, aspartate and glutamate, sphingolipid, and retinol metabolism which regulate those beneficial effects on risk factors for CVD (83).

In the present study white toast bread was used as control intervention, because this typical component of German breakfast is low in dietary fiber. As limitation for comparability the side dishes of toast like butter, sausages, or jam often contain more energy, fat, and carbohydrates than the side dishes for cereal flakes like fruits and yogurt. For standardization, subjects also received recipes for the breakfast with toast. The energy content was similar between the flakes and control recipes, but the content of carbohydrates and dietary fiber was lower in the control recipes (Table 2). Subjects did not have to cook exactly following the recipes as long they ate the 80 g of flakes or the four slices of white toast bread. In contrast to the nutritional value of the recipes, results from the FFP showed, that subjects significantly consumed more energy, carbohydrates, fat, oleic acid, SFA, MUFA, sodium, and minerals during the control intervention (Table 4). This may have contributed also to significantly increased TC and LDL cholesterol concentrations (Table 4) (84, 85). The increased intake of energy, carbohydrates, and fats during the control period might also result from a lower satiety because of the regular consumption of the toast bread (86).

The present study was conducted to study the effect of a regular consumption of traditional and roasted oat and barley flakes on blood lipids and glucose metabolism. The data show a small unfavorable effect of roasting on glucose metabolism as the significant decrease of AUC glucose after consumption of traditional oat flakes was not observed after test meal with roasted oat flakes. On the other side, advantages were detected for regularly intake of roasted barley flakes which resulted in significant decreases of body weight, BMI, as well as systolic and diastolic blood pressure. These reductions on cardiovascular risk factors were not identified after consumption of traditional barley flakes. We assume that the increased sensory quality by roasting improved compliance with the study intervention which results in these valuable effects. In this context, it must also be mentioned that the daily consumption of roasted barley flakes resulted in a significant decrease of AUC HDL cholesterol. This was not observed for traditional barley flakes.

To our knowledge, this is the first study, which directly compared the physiological effects of a regular consumption of traditional and roasted barley and oat flakes. Although the consumption of all cereal flakes reduced TC and LDL cholesterol concentrations significantly within 3 weeks after a daily consumption, TC and LDL cholesterol returned to basal values after 3 weeks of wash-out. This highlights the importance of a regular consumption of β-glucan-rich cereal flakes to achieve clinically relevant reductions of cardiovascular risk factors in the long-term. The improved sensory quality after roasting represents a promising alternative to increase consumption and long-term compliance (10, 11). Nevertheless, oat and barley flakes have a high susceptibility to oxidation, which may result in off-flavors. Roasted variants of flakes showed the same positive physiological effects as the traditional forms, but the improved sensory quality had to be ensured by an optimal storage at low moisture, low temperature, and low oxygen concentrations (10, 87). For the future, more studies are needed which specifically investigate the effects of molecular weight, viscosity, water-solubility, and the surrounding food matrix of roasted β-glucan-rich cereal flakes on glucose and lipid metabolism.

## 5. Conclusion

This study confirmed that the regular consumption of 80 g traditional and roasted barley and oat flakes daily for breakfast for 3 weeks effectively reduces fasting and postprandial TC and LDL cholesterol concentrations. The reducing effect on postprandial glucose response after consumption of roasted oat flakes was mitigated compared to traditional oat flakes. Roasting did not reduce the physiological effects on blood lipids. This indicates that processing like roasting with dry heat does not affect the health impact of β-glucan-rich roasted cereal flakes on lipid metabolism of moderately increased LDL cholesterol healthy subjects.

## Data availability statement

The raw data supporting the conclusions of this article will be made available by the authors, without undue reservation.

## Ethics statement

The study involving human participants was reviewed and approved by Ethical Commission of the Friedrich Schiller University, Jena. The patients/participants provided their written informed consent to participate in this study.

## Author contributions

MG, WS, SL, and CD: conceptualization, methodology, funding acquisition, and supervision. SRe, SH, and CD: validation and data curation. LW and SRe: formal analysis and statistical analysis. SRe, SH, MK, AK, SRu, and CD: investigation. SL, MK, and CD: resources. SRe: writing—original draft preparation. MG, SL, WS, and CD: writing—review and editing. SRe: visualization. MG, WS, and CD: project administration. All authors have read and agreed to the published version of the manuscript.

## Footnotes

1
https://www.gerstoni.de
