# Supplementary material for: Effect of a regular consumption of traditional and roasted oat and barley flakes on blood lipids and glucose metabolism–A randomized crossover trial
Source: Front Nutr. 2023 Feb 2;10:1095245. doi: 10.3389/fnut.2023.1095245 (PMC9932717; doi:10.3389/fnut.2023.1095245)
Supplement: Supplementary file 1 [file Data_Sheet_1.pdf]

Friedrich Schiller University Jena
Institute of Nutritional Sciences
Junior Research Group *Nutritional Concepts* (NuCo)
Dornburger Str. 27/29
07743 Jena

5-DAY FOOD FREQUENCY PROTOCOL

gLUCAn-Study

Subject number:______________

Start date:___________________

End date:____________________

Dear participant,

using this food frequency log, we want you to document, what you eat in **five consecutive days**.

Please note during filling out:

1. Please keep the food frequency protocol always with you (at home, in a restaurant, etc.). **Write down everything, you eat and drink**, also sauces, nuts, candies and little snacks during watching TV.

2. Please do not change your dietary habits during these 5 days. We want to record your usual dietary habits with this food frequency protocol.

3. Make a streak for the indicated portion size. The protocol should be kept continuously so that no differentiation of single days is necessary.

4. Please do not change the portion sizes.

5. If you do not find a consumed food in the table, please add it to ´other´. Please give a specification of the food item as well as the consumed amount.

If there are any upcoming questions, do not hesitate to contact us:

M. Sc. Sarah Reiners

Junior Research Group *Nutritional Concepts*
Institute for Nutritional Sciences
Dornburger Str. 25
07743 Jena
tel.: 03641 / 949726

email: sarah.reiners@uni-jena.de

Dr. Christine Dawczynski

Junior Research Group *Nutritional Concepts*
Institute for Nutritional Sciences
Dornburger Str. 29
07743 Jena
tel.: 03641 / 949656

email: christine.dawczynski@uni-jena.de

Quantity			Bread
	Pieces	45 g	Bun
	Pieces	50 g	Croissant (flaky pastry)
	Slices	45 g	Mixed wheat and rye bread
	Slices	45 g	Braided yeast bun
	Slices	10 g	Crisp bread
	Slices	20 g	White bread
	Pieces	55 g	Whole-grain roll
	Slices	50 g	Whole-grain bread
	Slices	35 g	Wheat bread
	Slices	10 g	Rusk
**Quantity**			**Sandwich filling**
	Teaspoons	5 g	Butter
	Teaspoons	5 g	Margarine, linoleic acid 30-50%
	Teaspoons	5 g	Margarine, linoleic acid >50%
	Portions	30 g	Blue cheese 50% FDM
	Tablespoons	30 g	Cream cheese
	Portions	30 g	Soft cheese 60-85% FDM
	Slices	30 g	Semi-hard cheese 30% FDM
	Slices	30 g	Semi-hard cheese 50% FDM
	Slices	30 g	Camembert 45% FDM
	Slices	30 g	Camembert 60-85% FDM
	Slices	25 g	Ham sausage
	Portions	25 g	Corned Beef
	Slices	20 g	Bologna
	Slices	30 g	Meat loaf
	Portions	30 g	Liver sausage
	Portions	30 g	Mettwurst
	Portions	30 g	Tea sausage spread
	Slices	20 g	Cervelat sausage
	Slices	15 g	Lachsschinken
	Slices	30 g	Wet-cured ham
	Portions	30 g	Pork belly
	Tablespoons	20 g	Blossom honey
	Tablespoons	20 g	Jam
	Tablespoons	20 g	Hazelnut spread
	Portions	30 g	Veggie pastry
**Quantity**			**Further supplements for breakfast**
	Pieces	55 g	Hen´s egg, cooked
	Tablespoons	4 g	Cornflakes
	Tablespoons	6 g	Cornflakes, roasted with sugar/honey
	Tablespoons	10 g	Cereal flakes
	Tablespoons	15 g	Muesli

Quantity			**Milk and dairy goods**
	Glasses	200 g	Buttermilk
	Cups	150 g	Yoghurt, partly skimmed
	Cups	150 g	Yoghurt, full-fat
	Cups	150 g	Yoghurt, low-fat with fruit preparation
	Cups	150 g	Yoghurt, full-fat with fruit preparation
	Glasses	200 g	Cow´s milk, low-fat (1,5 %)
	Glasses	200 g	Cow´s milk, full-fat (3,5 %)
	Glasses	200 g	Cow´s milk with cocoa/chocolate
	Tablespoons	20 g	Quark 10% FDM
	Tablespoons	20 g	Quark 20% FDM
	Tablespoons	10 g	Whipping cream 30 % fat
	Portions	12 g	Condensed milk 7.5 % fat
**Quantity**			**Fruits**
	Portions	125 g	Berries (blackberry, strawberry, blueberry, rawsberry, currant etc.)
	Portions	150 g	Grapes, fresh
	Portions	150 g	Pip fruit (apple, pear, quince etc.)
	Portions	150 g	Stone fruit (apricot, cherry, mirabelle, plum, peach etc.)
	Portions	120 g	Banana, fresh
	Portions	150 g	Tropical fruits (pineapple, kiwi, mango, passion fruit etc.)
	Portions	150 g	Citrus fruits (grapefruit, mandarin, melone, orange, lemon etc.)
	Portions	50 g	Raisins, dried fruit
**Quantity**			**Other**
	Pieces	50 g	Pickles
	Portions	100 g	Nuts
	Portions	100 g	Olives
	Cups	100 g	Peanuts (roasted and salted)
	Cups	50 g	Peanut flips
	Cups	30 g	Chips
	Portions	30 g	Saltsticks
**Quantity**			**Soups/Stews**
			as a starter
	Portions	200 g	Buoillon
	Portions	200 g	Soup, pale and thickened
	Portions	200 g	Tomato cream soup
	Portions	200 g	Goulash soup
			as the main course
	Portions	350 g	Pottage
	Portions	400 g	Potato soup
	Portions	450 g	Lentil stew

Quantity			Meat
	Portions	100 g	Minced meat of beef/pork, fresh
	Portions	200 g	Veal, medium-fat
	Portions	200 g	Beef, medium-fat
	Portions	200 g	Pork, medium-fat
	Portions	170 g	Pig´s offal, cooked
	Portions	170 g	Pork chop
	Portions	200 g	Pork schnitzel breaded, fried
	Portions	100 g	Canned sausage
	Portions	370 g	Fried chicken
	Portions	170 g	Poultry, cooked
**Quantity**			**Fish**
	Portions	175 g	Pollack, fried
	Portions	175 g	Plaice, fried
	Portions	175 g	Redfish, fried
	Portions	175 g	Pangas catfish, fried
	Portions	175 g	Trout, fried
	Portions	175 g	Salmon, fried
	Portions	175 g	Zander, fried
	Portions	175 g	Carp, steamed
	Portions	85 g	Tuna in water
	Portions	100 g	Fried herring
	Portions	125 g	Sardines in oil
	Portions	65 g	Canned herring
**Quantity**			**Garnish**
	Portions	200 g	Potatoes, peeled and cooked
	Portions	200 g	Potato dish, fried
	Portions	200 g	Mashed potatoes
	Pieces	100 g	Potato dumplings
	Pieces	75 g	Potato pancake
	Portions	180 g	Rice, cooked
	Portions	180 g	Brown rice, cooked
	Portions	180 g	Pasta, egg-free and cooked
	Portions	180 g	Wholegrain pasta, cooked
	Pieces	100 g	Bread dumpling
**Quantity**			**Sauces and fats**
	Portions	20 g	Marinade of vinegar
	Portions	75 g	Béchamel sauce
	Portions	100 g	Minced meat sauce
	Portions	75 g	Chasseur sauce
	Portions	75 g	Cheese sauce
	Portions	20 g	Barbecue sauce
	Portions	20 g	Tomato ketchup
	Teaspoons	6 g	Tomato purée
	Teaspoons	6 g	Mustard
	Tablespoons	12 g	Mayonnaise 80% fat
	Tablespoons	10 g	Dripping (animal fat)

	Tablespoons	10 g	Vegetable oils
**Quantity**			**Vegetable / Salads**
	Portions	60 g	Green salad with dressing
	Portions	180 g	Raw vegetable salad with cream sauce
	Portions	200 g	Leaf vegetables (celery, mangel, spinach, etc.)
	Portions	200 g	Green beans, fresh
	Portions	200 g	Fruit vegetables (aubergine, cucumber, pepper, tomato, courgette, etc.)
	Portions	200 g	Sweetcorn, cooked
	Portions	200 g	Cabbage (cauliflower, broccoli, white and red cabagge, kale, kohlrabi, sprouts, etc.)
	Portions	150 g	Sauerkraut
	Portions	200 g	Stalk and leek vegetables (fennel, leek, asparagus)
	Portions	200 g	Root vegetables (carrot, radish, Rettich, beetroot, turnips, celery, etc.)
	Portions	120 g	Mushrooms, cooked
**Quantity**			**Ready-made meal**
	Portions	250 g	Pasta salad with apples, tomatoes, and mayonnaise
	Portions	250 g	Sausage salad
	Portions	300 g	Greek salad
	Portions	300 g	Italien salad
	Pieces	100 g	Burger
	Pieces	120 g	Cheeseburger
	Pieces	200 g	Big Mac
	Portions	200 g	Swabian pockets
	Portions	300 g	Pancakes
**Quantity**			**Legumes**
	Portions	200 g	White beans, cooked
	Portions	200 g	Peas, cooked
	Portions	200 g	Lentils, cooked
**Quantity**			**Desserts / Cake / Candies**
	Portions	150 g	Custard
	Pieces	50 g	Ice cream
	Pieces	120 g	Fruit pie
	Pieces	150 g	Cream pie
	Pieces	60 g	Stirred cake
	Pieces	10 g	Cookie
	Pieces	20 g	Milk chocolate
	Pieces	10 g	Filled chocolates
	Pieces	3 g	Hard caramels
	Portions	50 g	Gumdrop
	Teaspoons	5 g	White sugar

Quantity			Beverages
	Cups	150 g	Coffee
	Cups	150 g	Black tea
	Glasses	200 g	Soda
	Glasses	200 g	Lemonade
	Glasses	200 g	Coke
	Glasses	200 g	Fruit juice
	Glasses	200 g	Fruit nectar
	Glasses	330 g	Alcohol-free beer
	Glasses	330 g	Pale ale
	Glasses	500 g	Wheat beer
	Glasses	200 g	White wine, semidry
	Glasses	200 g	Red wine
	Glasses	100 g	Sparkling wine
	Glasses	40 g	Liqueur
	Glasses	20 g	Brandies

Other

Quantity	Food item
