# Supplementary material for: Effect of a regular consumption of traditional and roasted oat and barley flakes on blood lipids and glucose metabolism–A randomized crossover trial
Source: Front Nutr. 2023 Feb 2;10:1095245. doi: 10.3389/fnut.2023.1095245 (PMC9932717; doi:10.3389/fnut.2023.1095245)
Supplement: Supplementary file 2 [file Table_1.DOCX]

Supplementary Material

# Supplementary Table

**Table S1.** Biochemical assays carried out in the Institute of Clinical Chemistry and Laboratory Diagnostics, University Hospital Jena, accredited according to DIN EN 17025.

| **Parameter** | **Method/Instrument** |
| --- | --- |
| *Plasma, serum* | |
| Total cholesterol, [mmol/l] | Abbott Architect CI 16200 analyzer (Abbott, Wiesbaden, Germany) |
| LDL cholesterol, [mmol/l] | Abbott Architect CI 16200 analyzer (Abbott) |
| HDL cholesterol, [mmol/l] | Abbott Architect CI 16200 analyzer (Abbott) |
| Triglycerides [mmol/l] | Abbott Architect CI 16200 analyzer (Abbott) |
| Glucose [mmol/l] | Abbott Architect CI 16200 analyzer (Abbott) |
| Insulin [mU/l] | Abbott Architect CI 16200 analyzer (Abbott) |
| Hemoglobin A_1c_ [%] | Tosoh HLC-723G11 (Sysmex, Norderstedt, Germany) |
| High-sensitive c-reactive protein [mg/l] | Abbott Architect CI 16200 analyzer (Abbott) |
| Erythrocytes [g/l] | XN 1000 (Sysmex) |
| Leukocytes [Gpt/l] | XN 1000 (Sysmex) |
| Thrombocytes [Gpt/l] | XN 1000 (Sysmex) |
| Hematocrit [%] | XN 1000 (Sysmex) |
| RDW [%] | XN 1000 (Sysmex) |
| Mean corpuscular hemoglobin (MCH) [fmol] | XN 1000 (Sysmex) |
| Mean corpuscular hemoglobin concentration (MCHC) [mmol/l] | XN 1000 (Sysmex) |
| Mean corpuscular volume, (MCV) [fl] | XN 1000 (Sysmex) |
| Ferritin [µg/l] | Abbott Architect CI 16200 analyzer (Abbott) |
| Transferrin [g/l] | Abbott Architect CI 16200 analyzer (Abbott) |
| Hemoglobin [mmol/l] | XN 1000 (Sysmex) |
